# Anti-obesity effects of the dual-active adenosine A_2A_/A_3_ receptor-ligand LJ-4378

**DOI:** 10.1038/s41366-022-01224-x

**Published:** 2022-09-27

**Authors:** Kyungmin Kim, Hyeonyeong Im, Yeonho Son, Minjae Kim, Sushil Kumar Tripathi, Lak Shin Jeong, Yun-Hee Lee

**Affiliations:** College of Pharmacy and Research Institute of Pharmaceutical Sciences, Seoul National University, Seoul, 08826 Korea

**Keywords:** Obesity, Drug discovery

## Abstract

**Background and objectives:**

A_2A_ adenosine receptor (A_2A_AR)-mediated signaling in adipose tissues has been investigated as a potential target for obesity-related metabolic diseases. LJ-4378 has been developed as a dual-acting ligand with A_2A_AR agonist and A_3_ adenosine receptor (A_3_AR) antagonist activity. The current study aimed to investigate the anti-obesity effects of LJ-4378 and its underlying molecular mechanisms.

**Methods:**

Immortalized brown adipocytes were used for in vitro analysis. A high-fat diet (HFD)-induced obesity and cell death-inducing DFFA-like effector A reporter mouse models were used for in vivo experiments. The effects of LJ-4378 on lipolysis and mitochondrial metabolism were evaluated using immunoblotting, mitochondrial staining, and oxygen consumption rate analyses. The in vivo anti-obesity effects of LJ-4378 were evaluated using indirect calorimetry, body composition analyses, glucose tolerance tests, and histochemical analyses.

**Results:**

In vitro LJ-4378 treatment increased the levels of brown adipocyte markers and mitochondrial proteins, including uncoupling protein 1. The effects of LJ-4378 on lipolysis of adipocytes were more potent than those of the A_2A_AR agonist or A_3_AR antagonist. In vivo, LJ-4378 treatment increased energy expenditure by 17.0% (*P* value < 0.0001) compared to vehicle controls. LJ-4378 (1 mg/kg, i.p.) treatment for 10 days reduced body weight and fat content by 8.24% (*P* value < 0.0001) and 24.2% (*P* value = 0.0044), respectively, and improved glucose tolerance in the HFD-fed mice. LJ-4378 increased the expression levels of brown adipocyte markers and mitochondrial proteins in interscapular brown and inguinal white adipose tissue.

**Conclusion:**

These findings support the in vivo anti-obesity effects of LJ-4378, and suggest a novel therapeutic approach to combat obesity and related metabolic diseases.

## Introduction

Obesity is defined as the excess accumulation of fat, which is a major risk factor for various metabolic diseases, such as type 2 diabetes [1]. Adipose tissue can be categorized into brown adipose tissue (BAT) and white adipose tissue (WAT) [2]. While BAT contains numerous mitochondria and is responsible for non-shivering thermogenesis, WAT specializes in fat storage and mobilization [3]. Thus, the activation and recruitment of BAT is a strategy to increase energy expenditure, which can ultimately be developed as a potential anti-obesity target [4].

The pharmacological stimulation of adenosine receptor signaling has received significant attention as a potential target for improving metabolic health [5]. Adenosine receptors can be categorized into four types: A_1_, A_2A_, A_2B_, and A_3_ [6]. The A_1_ adenosine receptor (A_1_AR) and A_3_ adenosine receptor (A_3_AR) coupled to G_i_ proteins inhibit the adenylate cyclase (AC)-mediated cAMP production and cAMP-dependent protein kinase (PKA) signaling. In contrast, the A_2A_ adenosine receptor (A_2A_AR) and A_2B_ adenosine receptor (A_2B_AR) are coupled to G_s_ proteins, facilitating AC activity [7].

Among adenosine receptors, A_2A_AR is more abundantly expressed in BAT than in WAT [8]. A_2A_AR signaling is necessary for the complete physiological function of BAT, and its anti-obesity effects have been supported by several studies [9]. Although A_3_AR is expressed in WAT [8], its regulatory roles in adipocyte metabolism are not yet fully understood.

In this study, we investigated the anti-obesity effects of LJ-4378, a dual-acting ligand with A_2A_AR agonist and A_3_AR antagonist activities [10], using a high-fat diet (HFD)-induced obesity mouse model. The in vitro effects of LJ-4378 on lipolysis and mitochondrial metabolism were evaluated using immortalized brown adipocytes and compared to those of the A_2A_AR agonist and A_3_AR antagonist.

## Methods

### Cell culture

Brown adipocytes were differentiated from immortalized preadipocytes obtained from interscapular BAT of mice as previously described [11]. Cells were cultured in Dulbecco’s modified Eagle’s medium (Welgene, LM001-07, St. Louis, MO, USA) containing 10% fetal bovine serum (FBS, Gibco, 16000044, Waltham, MA, USA) and 1% Penicillin Streptomycin (Welgene, LS202-02, Waltham, MA, USA) at 37 °C in a humidified atmosphere with 5% CO_2_. When the cells reached about 90% confluency, the cells were exposed to a differentiation medium supplemented with 2.5 mM isobutyl methylxanthine (IBMX, Cayman, I5879, Ann Arbor, MI, USA), 0.125 mM indomethacin (Cayman, 70270, Ann Arbor, MI, USA), 1 μM dexamethasone (Cayman,11015, Ann Arbor, MI, USA), 1 μg/mL insulin (Sigma, I9278, St. Louis, MO, USA), and 1 nM triiodothyronine (T3, Cayman, 6028, Arbor, MI, USA) for 3 days for adipogenic differentiation. Then the cells were maintained in a growth media with 1 μg/mL insulin and 1 nM T3 (triiodothyronine) for 3 days. Intracellular cAMP levels were measured using the Direct cAMP ELISA Kit (Enzo Life Science, ADI-901-066A, New York, NY, USA) in accordance with the manufacturer’s protocol.

3T3-L1 cells obtained from ATCC (Manassas, VA, USA) were cultured in growth medium (Dulbecco’s Modified Eagle’s Medium (Welgene, LM001-07, St. Louis, MO, USA) supplemented with 10% FBS and 1% Penicillin Streptomycin)) at 37 °C in a humidified atmosphere with 5% CO_2_ [11]. Cells were then exposed to adipogenic differentiation medium (growth medium supplemented with 0.125 mM indomethacin (Cayman, 70270, Ann Arbor, MI, USA), 2.5 mM isobutyl methylxanthine (IBMX, Cayman, I5879, Ann Arbor, MI, USA), 1 µM dexamethasone (Cayman,11015, Ann Arbor, MI, USA), 1 μg/mL insulin (Sigma, I9278, St. Louis, MO, USA), and 1 nM triiodothyronine (T3, Cayman, 6028, Arbor, MI, USA)) for 3 days. For maintenance of differentiation, cells were exposed to a maintenance medium (growth medium supplemented with 1 µg/mL insulin and 1 nM triiodothyronine) for 3 days. For siRNA knockdown, fully differentiated 3T3L1 adipocytes were transfected with Negative Control siRNA (Bioneer, SN-1013, Daejeon, Republic of Korea) or A_2A_AR siRNA (Bioneer, 1211, Daejeon, Republic of Korea) using INTERFERin (Polyplus, 409-10, Florida, NY, USA) according to the manufacturer’s protocol. For in vitro experiments, cells were randomly assigned to each experimental group.

The LJ-4378, LJ-4433, and LJ-529 were synthesized as described previously [10, 12, 13]. CGS21680 (Cayman, 124431-80-7, Ann Arbor, MI, USA) was used for experiments.

### Oxygen consumption rate (OCR)

XFp Analyzers were used to measure oxygen consumption rate (OCR). Cells were incubated in XF DMEM base medium (Agilent, 103575-100, pH 7.4, Cedar Creek, Texas, USA) supplemented with 4 mM L-glutamine (Sigma, G8540, St. Louis, MO, USA) and 25 mM D-glucose (Sigma, G7021, St. Louis, MO, USA) at 37 °C for the measurement. XFp Cell Mito Stress Test Kit (Agilent, 103010-100, Cedar Creek, Texas, USA) was sequentially prepared with the following optimal final concentrations: 2.5 μM oligomycin, 0.5 μM FCCP, and 0.5 μM rotenone/antimycin A, and basal, maximal, proton leak and were calculated previously described [14].

### Immunocytochemistry

Cells were exposed to MitoTracker^™^ Red CMXRos (1:3000, Invitrogen, M7512, Waltham, MA, USA) for 15 min at 37 °C for mitochondria staining, then fixed with 4% paraformaldehyde (PFA, Sigma, 158127, St. Louis, MO, USA) as previously described [15]. Images were obtained using LSM800 confocal microscope (Zeiss, Germany) and analyzed with Zen software (version 3.0).

### Cytotoxicity assay

EZ-CYTOX (DoGEN, EZ-3000, Seoul, Republic of Korea) was used to evaluate the cell viability, according to the manufacturer’s instructions. Absorbance was measured at 450 nm using a microplate reader (Thermo MULTISKAN GO, 8816-2015, Waltham, MA, USA).

### Analysis of glycerol and free fatty acid (FFA) assay

Glycerol and free fatty acid (FFA) levels in media were measured using glycerol reagent (Sigma-Aldrich, F6428, St. Louis, MO, USA) and NEFA reagents (WAKO, 436-91693, Osaka, Japan) following the manufacturer’s product protocol as previously described [16].

### Animals

Animal experiments were performed following approved protocols by Institutional Animal Care and Use Committees of Seoul National University (SNU-201107-1, SNU-201221-3). C57BL/6 male mice and CIDEA reporter mice [17] were used (8-week-old, male). Mice were housed at 12 h-light/12 h-dark cycle condition with free access to a normal chow diet (NCD, Purina Lab, 38057, protein: 24.52% calories, carbohydrates: 63.07% calories, fat: 12.41% calories, Seongnam, Republic of Korea) and water at 22 ± 1 °C. For the diet-induced obesity model, 8-week-old male mice were fed a HFD (Research Diets, D12492, protein: 20% kcal, carbohydrate: 20% kcal, fat: 60% kcal, New Brunswick, NJ, USA) for 8 weeks. Mice were treated either with LJ-4378 (1 mg kg^−1^ day^−1^) or vehicle intraperitoneally for 10 days [8]. LJ-4378 was dissolved in dimethylsulfoxide (DMSO) and diluted in sterile 0.9% saline (0.2% DMSO as the final concentration).

Indirect calorimetry was performed to measure expenditure (EE), VO_2,_ VCO_2_, activity and food intake using PhenoMaster (TSE Systems, Bad Homburg, Germany). Body composition was measured by nuclear magnetic resonance scanning EchoMRI-700 (Echo Medical Systems, India).

Mice were randomly assigned to experimental groups, and experimental groups of mice were not blinded.

### Western blot analysis and quantitative PCR

Western blot analysis and qPCR were conducted, as described previously [17]. Primers used for the qPCR analyses are listed in Supplementary Table S1. Primary antibodies used for western blot are summarized in Supplementary Table S2. Anti-rabbit horseradish peroxidase antibodies (1:3000, Thermo Fisher, 31460, Waltham, MA, USA) and anti-mouse horseradish peroxidase antibodies (1:3000, Jackson, 115-035-174, USA) were used for secondary antibodies. All antibodies were diluted in blocking buffer (5% skim milk or 5% bovine serum albumin in TBST). Western blot images were acquired using the Fusion Solo chemiluminescence imaging system (Vilber Lourmat, France) and analyzed with EvolutionCapt software (version 17.03). Images of the whole western blot membrane are provided in Supplementary Data. NIH ImageJ software was used for the quantification.

### Histology

Adipose tissues were fixed using 10% formalin (Sigma, St. Louis, MO, USA) and then embedded in paraffin blocks. The paraffin sections were stained with hematoxylin/eosin (H&E) (BBC biochemical, McKinney, TX, USA) as described previously [14].

### Glucose tolerance test

For the glucose tolerance test, mice were intraperitoneally injected with 20% D-Glucose (1 g kg^−1^, Sigma-Aldrich, G7021, St. Louis, MO, USA) as described previously [18]. A glucose meter (Gluco Dr. Top, allmedicus, AGM-4100, Anyang, Republic of Korea) was used to measure glucose concentration.

### TTC analysis

Ex vivo electron transport activity related to mitochondrial oxidative phosphorylation was evaluated by monitoring the reduction of 0.1% triphenyltetrazolium chloride (TTC, Sigma, T8877, St. Louis, MO, USA) as described previously [16].

### Bioluminescence and fluorescence imaging

In vivo bioluminescence was detected as previously described using an optical imaging device (Ami-X, Spectral Instruments Imaging) [17]. To detect in vivo bioluminescence signal, mice were i.p. injected with D-luciferin (150 mg kg^−1^, Goldbio, St. Louis, MO, USA). Aura Software (Spectral Instruments Imaging, Version 2.2.1.1) was used for quantification. Also, ex vivo imaging, tissues were collected from the CIDEA reporter mice treated with D-luciferin (150 mg kg^−1^, Goldbio, St. Louis, MO, USA). During imaging, the isolated tissues were maintained in 12 well plates containing D-luciferin (300 μg/ml).

### Statistical analysis

Sample sizes were determined based on reproducibility and statistical significance. Prism 7 software (GraphPad Software, USA) was used for statistical analysis. Data were shown as mean ± SEM. An unpaired *t-*test was used between two groups to measure statistical significance. Data were normally distributed with equal variances between sample groups. No data were excluded from in vitro and in vivo experiments.

## Results

### LJ-4378 treatment upregulates lipolysis and mitochondrial content in a dose-dependent manner in vitro

**Fig. 1:**
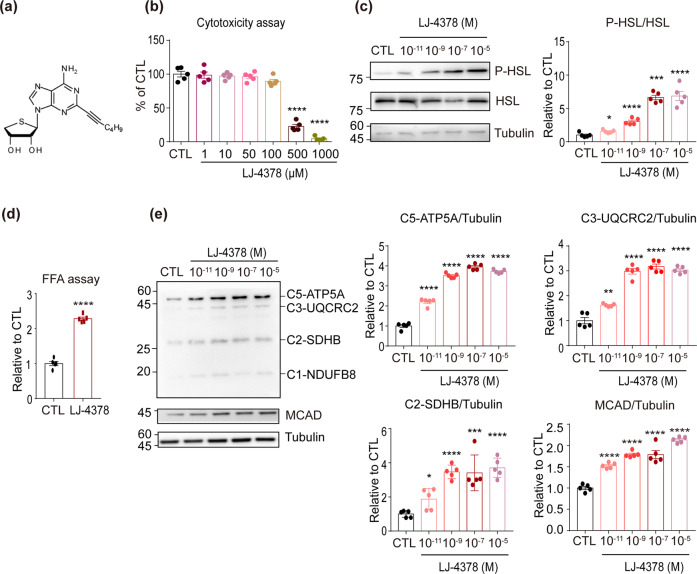
LJ-4378 treatment increases lipolysis and mitochondrial content of adipocytes in a dose-dependent manner. **a** The chemical structure of LJ-4378; (2R,3S,4R)-2-(6-Amino-2-(hex-1-ynyl)-9H-purin-9-yl)- tetrahydrothiophene-3,4-diol. **b** Cytotoxicity effects of LJ-4378 on brown adipocytes (BAs) at indicated concentration (24 h treatment, *n* = 5, means ± SEM, ****p < 0.0001). **c** Immunoblot analysis of hormone-sensitive lipase (HSL) and phosphorylated HSL (p-HSL, serine 660) of BAs treated with LJ-4378 for 4 h (*n* = 5, means ± SEM, *p < 0.05, ***p < 0.001, ****p < 0.0001). **d** Free fatty acid (FFA) levels in conditioned media of BAs treated with 0.1 μM LJ-4378 for 24 h (*n* = 5, means ± SEM, ****p < 0.0001). **e** Immunoblot analysis of BAs treated with LJ-4378 at indicated concentration for 48 h (*n* = 5, means ± SEM, *p < 0.05, **p < 0.01, ***p < 0.001, ****p < 0.0001).

LJ-4378 is a dual-acting ligand with A_2A_AR agonist and A_3_AR antagonist activities (Fig. 1a) [10]. The cytotoxicity assay of LJ-4378 indicated that concentrations below 100 μM did not affect the brown adipocyte viability (Fig. 1b).

As A_2A_AR activates PKA signaling in adipocytes, the lipolytic effects of LJ-4378 were examined in brown adipocytes differentiated from immortalized brown pre-adipocytes. Immunoblotting analysis showed that LJ-4378 upregulated the protein levels of phosphorylated hormone-sensitive lipase (P-HSL) at serine 660 in a dose-dependent manner (Fig. 1c). Consistently, FFA levels were also increased by LJ-4378 (Fig. 1d: 2.29 ± 0.05 folds increase).

In addition, the levels of mitochondrial proteins involved in oxidative metabolism increased with an increase in the dose of LJ-4378 (Fig. 1e). Based on these dose-response assessments, a concentration of 0.1 μM was selected for all subsequent experiments.

### Lipolytic effects of LJ-4378 are more potent than those of the A_2A_AR agonist or A_3_AR antagonist

**Fig. 2:**
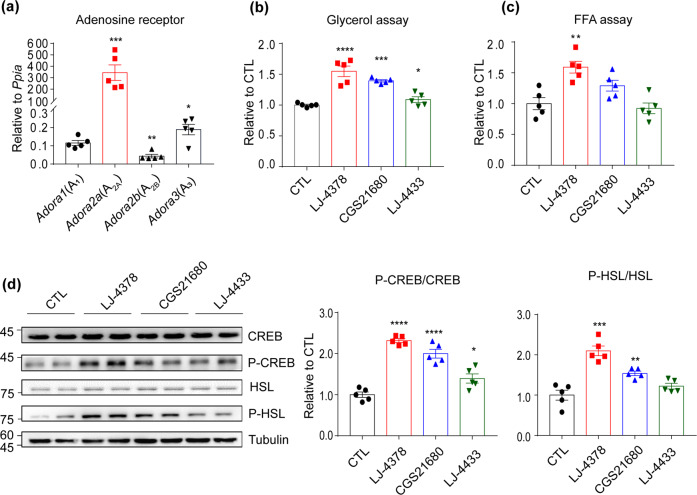
Lipolytic effects of LJ-4378 are more potent than those of A_2A_ agonist or A_3_ antagonist treatment. **a** qPCR analysis of expression levels of *Adora1* (A_1_), *Adora2a* (A_2A_), *Adora2b* (A_2B_), and *Adora3* (A_3_) adenosine receptors of BAs (*n* = 5, means ± SEM, *p < 0.05, **p < 0.01, ***p < 0.001). **b**, **c** Glycerol and FFA levels in conditioned media of BAs treated with LJ-4378 (0.1 μM), A_2A_ agonist (CGS21680, 0.1 μM), or A3 antagonist (LJ-4433, 0.1 μM) for 24 h (*n* = 5, means ± SEM, *p < 0.05, **p < 0.01, ***p < 0.001, ****p < 0.0001). **d** Immunoblot analysis of BAs treated with LJ-4378 (0.1 μM), A_2A_ agonist (CGS21680, 0.1 μM), or A_3_ antagonist (LJ-4433, 0.1 μM) for 24 h. (*n* = 5, means ± SEM, *p < 0.05, **p < 0.01, ***p < 0.001, ****p < 0.0001).

Quantitative polymerase chain reaction (qPCR) analyses were performed to determine the adenosine receptor expression levels in brown adipocytes differentiated from immortalized brown pre-adipocytes. A_2A_AR showed the highest expression, and A_3_AR showed the second highest expression among all the adenosine receptors examined (Fig. 2a). The lipolytic effect of LJ-4378 was compared to that of the A_2A_AR agonist (CGS21680) and A_3_AR antagonist (LJ-4433). In both the glycerol and FFA assays, LJ-4378 treatment exhibited the greatest increase compared to CGS21680 and LJ-4433 (Fig. 2b, c). Immunoblotting analysis further confirmed the upregulation of the expression levels of PKA downstream proteins, including phosphorylated cAMP response element-binding protein (P-CREB) and P-HSL (Fig. 2d). Consistently, LJ-4378 increased intracellular cAMP levels in BAs (Supplemental Fig. S1). Moreover, immunoblot analysis indicated that A_3_AR agonist (LJ-529) treatment reduces phosphorylation levels of PKA downstream proteins (Supplementary Fig. S2).

Next, we examined the effects of A_2A_AR knockdown on LJ-4378 induced lipolysis. A_2A_AR siRNA achieved nearly 70% knockdown of the A_2A_AR expression in 3T3L1 cells (Supplementary Fig. S3). We confirmed that LJ-4378 treatment increased phosphorylated hormone-sensitive lipase (P-HSL) levels in 3T3L1 cells. A_2A_AR knockdown significantly suppressed LJ-4378-induced phosphorylation of HSL (Supplementary Fig. S3).

### LJ-4378 increases mitochondrial content and metabolism in brown adipocytes

**Fig. 3:**
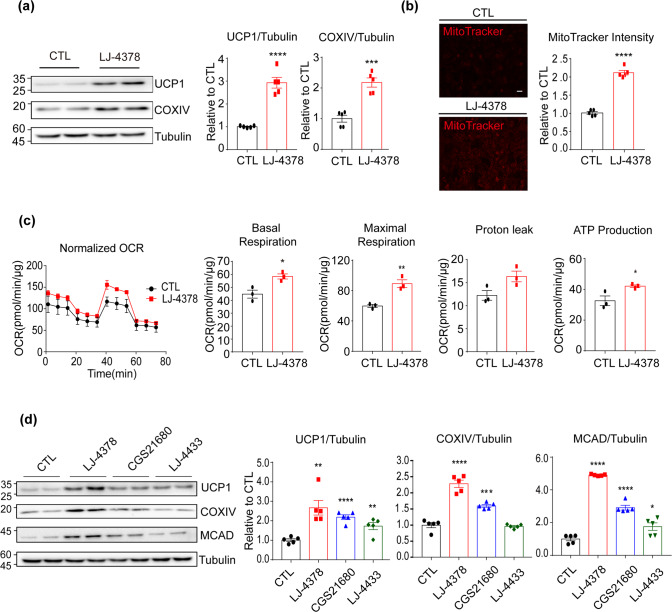
In vitro LJ-4378 treatment increases mitochondrial content and activity in adipocytes. **a** Immunoblot analysis of BAs treated with 0.1 μM LJ-4378 for 24 h (*n* = 5, means ± SEM, ***p < 0.001, ****p < 0.0001). **b** Representative images of MitoTracker staining of BAs treated with vehicle (CTL) and 0.1 μM LJ-4378 (*n* = 5, size bar = 20 μm). **c** OCR measurement of BAs treated with 0.1 μM LJ-4378 for 24 h (*n* = 3, means ± SEM, *p < 0.05, **p < 0.01). **d** Immunoblot analysis of BAs treated with LJ-4378 (0.1 μM), A_2A_ agonist (CGS21680, 0.1 μM), or A_3_ antagonist (LJ-4433, 0.1 μM) for 24 h (*n* = 5, means ± SEM, *p < 0.05, **p < 0.01, ***p < 0.001, ****p < 0.0001).

Next, the effects of LJ-4378 on the mitochondrial content and metabolic activity of brown adipocytes were evaluated. The expression levels of the brown adipocyte marker, uncoupling protein 1 (UCP1), and mitochondrial protein, cytochrome c oxidase subunit 4I1 (COXIV), were significantly increased by 2.67 ± 0.38 folds and 2.29 ± 0.11 folds, respectively, after LJ-4378 treatment (Fig. 3a). MitoTracker staining also demonstrated that LJ-4378 increased mitochondrial membrane potential by 2.12 ± 0.06 folds in brown adipocytes (Fig. 3b). The OCRs of basal, maximal, and ATP production-related respiration were increased by LJ-4378 (Fig. 3c). Furthermore, we compared the effects of LJ-4378 on the mitochondrial content with those of CGS21680 and LJ-4433. While both CGS21680 and LJ-4433 increased UCP1, COXIV, and MCAD levels, greater effects were observed in LJ-4378 (Fig. 3d).

### LJ-4378 increases the mitochondrial activity in adipose tissues and energy expenditure in vivo

**Fig. 4:**
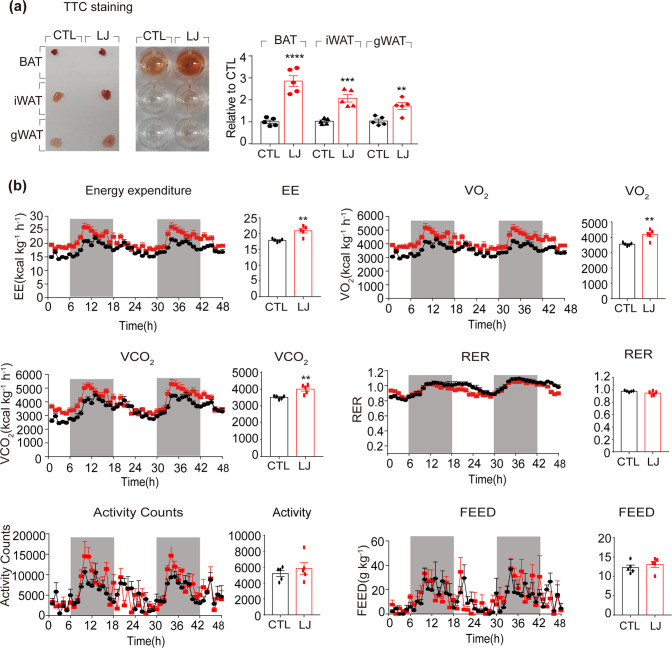
In vivo LJ-4378 treatment increases the mitochondrial oxidative metabolism of adipose tissue and energy expenditure. Mice were treated with vehicle (CTL), LJ-4378 (LJ, i.p., 1 mg kg^−1^/day) for 10 days. **a** 2,3,5-Triphenyltetrazolium chloride (TTC) staining of BAT, iWAT, and gWAT (*n* = 5, means ± SEM, **p < 0.01, ***p < 0.001, ****p < 0.0001). **b** Indirect calorimetry analysis of EE (energy expenditure), VO_2_ (rate of oxygen consumption), VCO_2_ (rate of carbon dioxide production), RER (respiratory exchange ratio), Activity Counts, food consumption (FEED) (*n* = 5, means ± SEM, **p < 0.01).

The effects of LJ-4378 were investigated in vivo. In situ staining with triphenyltetrazolium chloride (TTC), a redox indicator, demonstrated that LJ-4378 upregulated mitochondrial activity in all adipose tissue depots (Fig. 4a). Indirect calorimetric analysis further indicated that LJ-4378 treatment increased oxygen consumption by 17.8%, thereby increasing energy expenditure by 17.0%, while activity and food intake were unaffected (Fig. 4b).

### LJ-4378 treatment increases the browning of adipose tissues in vivo

**Fig. 5:**
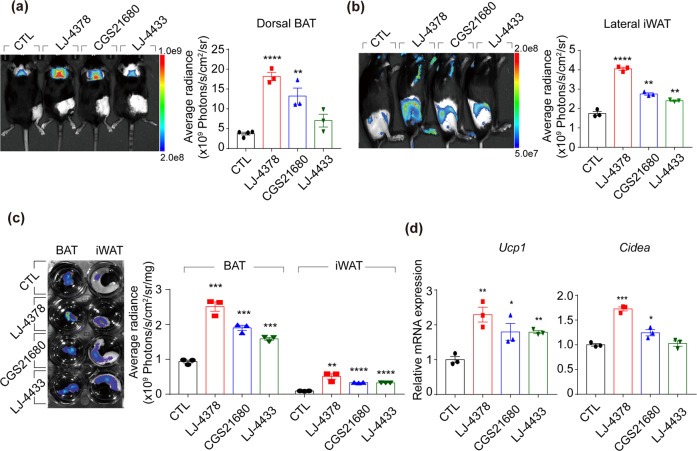
CIDEA reporter mouse study demonstrates browning effects of LJ-4378. Mice were treated with vehicle (CTL), LJ-4378 (LJ, 1 mg kg^−1^/day), A_2A_ agonist (CGS21680, 1 mg kg^−1^/day), or A_3_ antagonist (LJ-4433, 1 mg kg^−1^/day) for 10 days. **a**, **b** Representative bioluminescence of dorsal and lateral positioning (*n* = 3, means ± SEM, **p < 0.01, ****p < 0.0001). **c** A representative ex vivo fluorescence image of BAT and iWAT and quantification of fluorescence intensity (*n* = 3, means ± SEM, **p < 0.01, ***p < 0.001, ****p < 0.0001). **d** qPCR analysis of *Ucp1* and *Cidea* mRNA expression level of brown adipose tissue (BAT) (*n* = 3, means ± SEM, *p < 0.05, **p < 0.01, ***p < 0.001).

The effects of LJ-4378 on adipose tissue browning were examined in the cell death-inducing DFFA-like effector A (CIDEA) reporter mice. CIDEA is a marker of brown adipocytes, and this reporter system enables non-invasive monitoring of CIDEA expression based on bioluminescence signals. LJ-4378 significantly increased the bioluminescence intensities of BAT and inguinal white adipose tissue (iWAT) (Fig. 5a, b). In particular, there was a significant increase in iWAT compared with CGS21680 and LJ-4433. In line with the in vivo bioluminescence results, LJ-4378 treatment resulted in the most significant increase in the ex vivo luminescence signals of BAT and iWAT (Fig. 5c).

The mRNA levels of brown adipocyte markers were also examined and it was demonstrated that LJ-4378 resulted in the greatest increase in gene expression related to browning, including *Ucp1* and *Cidea* in BAT, among the adenosine receptor modulators (Fig. 5d).

### LJ-4378 protects mice against diet-induced obesity

**Fig. 6:**
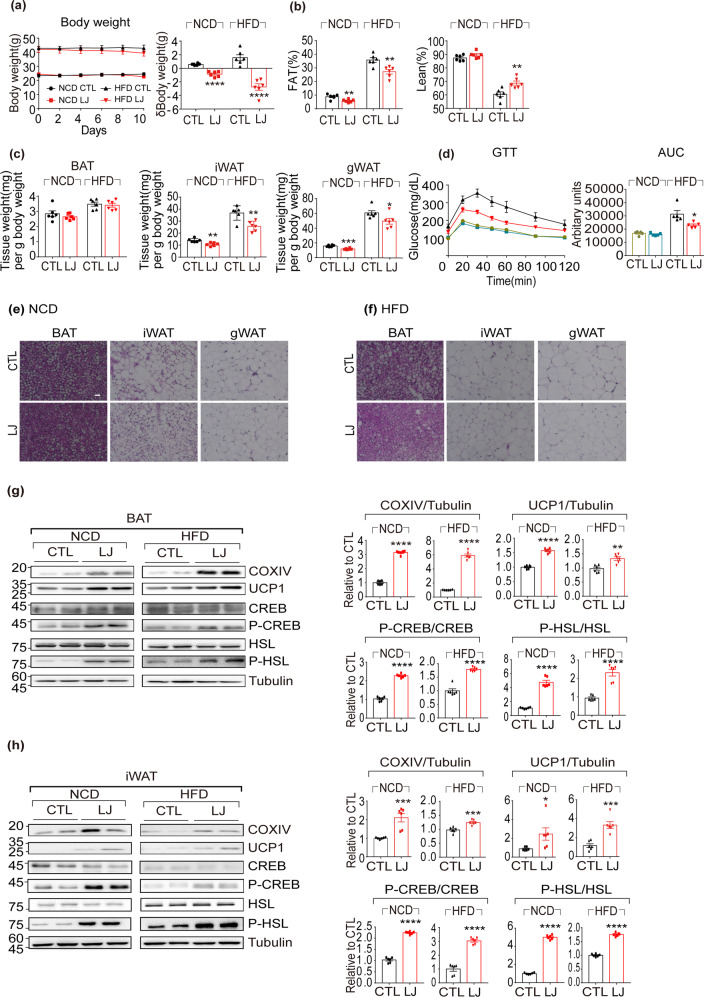
In vivo LJ-4378 treatment protects mice from high-fat diet-induced obesity by activation of the PKA signaling pathway in adipose tissue. Normal chow diet (NCD) and high-fat diet (HFD)-fed mice were treated with LJ-4378 (LJ, 1 mg kg^−1^/day) or vehicle controls (CTL) for 10 days. **a** Body weight changes (*n* = 6). **b** Body composition of fat percentage and lean percentage (*n* = 6, means ± SEM, **p < 0.01). **c** Tissue weight (mg) per g body weight of BAT, iWAT, gWAT (*n* = 6, means ± SEM, *p < 0.05, **p < 0.01). **d** Glucose tolerance test and area under the curve (AUC) (*n* = 5, means ± SEM, ^*^p < 0.05). **e**, **f** Hematoxylin and eosin (H/E) staining of paraffin sections of BAT, iWAT, and gWAT (size bar = 10 μm). **g** Immunoblot analysis in BAT (*n* = 6, means ± SEM, **p < 0.01, ****p < 0.0001). **h** Immunoblot analysis in iWAT. Tubulin was the loading control. (*n* = 6, means ± SEM, *p < 0.05, ***p < 0.001, ****p < 0.0001).

We then examined whether the lipolysis and browning effects of LJ-4378 would have an in vivo anti-obesity effect using an HFD-induced obesity mouse model. LJ-4378-treated HFD-fed mice showed 8.24% and 24.18% reduction in body weight and adiposity, respectively (Fig. 6a, b). Moreover, the iWAT and gonadal white adipose tissue (gWAT) weights were significantly decreased by LJ-4378 treatment in HFD-fed mice (Fig. 6c). LJ-4378 improved glucose tolerance in the HFD-fed mice (Fig. 6d). In addition, the reduced size of adipocytes in iWAT and gWAT was observed in H&E-stained sections (Fig. 6e, f and Supplementary Fig. S4).

Next, we examined the expression levels of mitochondrial proteins and PKA downstream markers in adipose tissue. LJ-4378 upregulated UCP1, COXIV, P-CREB, and P-HSL in BAT and iWAT of HFD-fed mice (Fig. 6g, h). However, LJ-4378 treatment did not significant effects on mitochondrial protein expression in muscle (Supplementary Fig. S5).

qPCR analysis of expression levels of genes involved in inflammatory responses demonstrated that LJ4378 treatment increased the anti-inflammatory markers (*Arg1* in iWAT and *Il10* in gWAT). By contrast, a pro-inflammatory marker Tnfa was reduced in both iWAT and gWAT (Supplementary Fig. S6).

## Discussion

Extracellular adenosine is a potent endogenous signaling molecule that regulates multiple physiological and pathological events via four types of adenosine receptors [9]. Studies using transgenic mice with altered adenosine receptor expression further support that adenosine exerts multiple effects on energy metabolism [19, 20]. Thus, targeting adenosine receptors may have translational potential for the treatment of metabolic diseases. This study investigated the effects of a dual ligand with A_2A_AR agonist and A_3_AR antagonist activities on adipocyte metabolism.

Previous studies demonstrated that A_2A_AR agonist treatment has anti-obesity effects partly via the activation of BAT thermogenesis and energy expenditure [8] and protected against hypertensive cardiac remodeling by facilitating the secretion of BAT-derived fibroblast growth factor 21, so-called “batokine” [21]; however, its crosstalk or synergistic effects with the activation of other adenosine receptors on adipocyte metabolism have not been fully investigated. While the stimulation of G_αs_-coupled A_2A_AR activates AC and cAMP–PKA signaling pathways, the activation of G_αi_-coupled A_3_AR inhibits AC, opposing the effects of A_2A_AR signaling. Thus, we hypothesized that the dual ligand with A_2A_AR agonist and A_3_AR antagonist activities could synergistically activate PKA-dependent lipolysis and thermogenesis in adipocytes.

This study demonstrated that LJ-4378 treatment increased the mitochondrial content and activity in adipocytes, and its effects were more potent than those of the A_2A_AR agonist or A_3_AR antagonist. In vivo, LJ-4378 treatment increased energy expenditure and the expression of the brown adipocyte marker CIDEA, which was determined by non-invasive imaging of bioluminescence signaling in CIDEA reporter mice. In vivo, LJ-4378 treatment reduced body weight and fat content and improved glucose tolerance in HFD-fed mice, indicating its anti-obesity effects.

qPCR analysis in the current study indicated that A_2A_AR and A_3_AR are the major subtypes of adenosine receptors expressed in adipocytes. In contrast, the expression levels of A_2B_AR in adipocytes were lower than those of other adenosine receptors [22]. A_2B_AR is expressed mainly in the muscle and BAT, and the activation of A_2B_AR exerts anti-aging and anti-obesity effects [22]. Interestingly, A_2B_AR has a permissive role in A_2A_-mediated lipolysis by forming heterodimers of the two receptors [22]. In contrast, A_1_ receptors, which are coupled to G_αi_, inhibit AC activity and are highly expressed in human white adipocytes [9]. A_1_ARs have been characterized as lipolysis inhibitors [9] that can promote insulin sensitivity in adipose tissue by reducing circulating FFA and triglyceride levels [23]. Moreover, A_1_AR signaling promotes lipogenesis and modulates inflammation, as shown in a A_1_AR knockout mouse study [24]. Further investigation is required to understand the contribution of individual and interactive adenosine receptor signaling to the development of novel therapeutics that target adenosine signaling to efficiently activate lipid catabolism and restore insulin sensitivity in adipocytes.

In addition to its effects on lipolysis, A_2A_AR activation exerted anti-inflammatory effects in a mouse model of diet-induced obesity [25]. In our previous study, LJ-4378 demonstrated in vivo anti-inflammatory effects in a rodent model of carrageenan-induced paw edema [10]. Although we focused on the thermogenic and lipolytic effects of the dual ligands, further studies are warranted to determine the effect of LJ-4378 on the polarization of adipose tissue macrophages and HFD-induced inflammation.

Other G_s_-coupled receptors have been investigated for their role in the activation of BAT thermogenesis. Although β3-adrenergic receptor-mediated signaling pathways are canonical pathways that activate the thermogenic function of BAT, the effects of β3-selective agonists in humans were unexpectedly low in clinical trials. Adenosine treatment inhibits isoproterenol-mediated lipid metabolism in brown adipocytes of hamsters, which contradicts the results obtained from mouse studies [26, 27]. However, these discrepancies might be due to the species-specific expression and activity of adenosine receptor isotypes [8]. In this regard, understanding the species-specificity of receptor expression and activity is a prerequisite for translational approaches.

The effects of adenosine signaling in a broad range of tissues may limit the clinical application of adenosine receptor ligands. For example. A_2A_ARs play important roles in the pathogenesis of hepatic fibrosis and cirrhosis [28, 29]. The limitation of current study is that the role of non-adipose tissues in mediating the in vivo effects of LJ-4378 cannot be ruled out until pharmacokinetic-pharmacodynamic studies and tissue-specific knockout studies are completed. Furthermore, tissue- or cell-type-specific targeting of pharmacological modulators of adenosine receptor signaling is necessary to develop therapeutic tools without potential adverse effects.

## Conclusions

In summary, our data demonstrate that the simultaneous regulation of adenosine A_2A_AR and A_3_AR activity has in vivo anti-obesity effects. These findings may aid in the development of a novel therapeutic approach for combating obesity and related metabolic diseases.

## Supplementary information

Supplemental Materials

## Data Availability

All data generated or analyzed during this study are included in this published paper and its supplementary information files.
