# Supplementary material for: Anti-obesity effects of the dual-active adenosine A2A/A3 receptor-ligand LJ-4378
Source: Int J Obes (Lond). 2022 Sep 27;46(12):2128–36. doi: 10.1038/s41366-022-01224-x (PMC9678795; doi:10.1038/s41366-022-01224-x)
Supplement: Supplementary file 1 — Supplemental Materials [file 41366_2022_1224_MOESM1_ESM.pdf]

Supplementary Table S1. qPCR primers

Genes	Forward (5′→3′)	Reverse (5′→3′)
Ucp1	TGG CCT CTC AGT GGA TGT G	CGT GGT CTC CCA GCA TAG AAG
Cidea	TGC TCT TCT GTA TCG CCC AGT	GCC GTG TTA AGG AAT CTG CTG
Ppia	GTG GTC TTT GGG AAG GTG AA	TTA CAG GAC ATT GCG AGC AG
Adora1	TGG CTC AGT TCG TGC ATC AT	ATC CTT ACC CAT CGA CTG CC
Adora2a	CAG AGA AGG GAA GCA ATC GCA	ACC ATG ACT TCT ACA GGG GGT
Adora2b	GCGTCCCGCTCAGGTATAAA	CAATGCCAAAGGCAAGGACC
Adora3	AGT AAG AAC GGT GGC CCT CT	ATC CCT GCC TTC CCA TTA ACC
Arg1	GGC TGG TGT GGT GGC AGA GG	CCT GGC GTG GCC AGA GAT GC
Il10	GGC AGA GAA GCA TGG CCC AGA	TCA CCT GCT CCA CTG CCT TGC
Tnfα	GAT CGG TCC CCA AAG GGA TG	CCA CTT GGT GGT TTG TGA GTG
Il12b	GGG AGC TGG AGA AAG ACG TTT	TGG TCT GAG GTC CAG GTG AT

Supplementary Table S2. Primary antibodies used for western blots

Antibody	Host	Company	Catalog #	Dilution
UCP1	Rabbit	Alpha diagnostic	UCP11-A	1:1000
COXIV	Rabbit	Cell signaling	4850	1:1000
HSL	Rabbit	Cell signaling	4107	1:1000
Phospho-HSL	Rabbit	Cell signaling	45804	1:1000
CREB	Rabbit	Cell signaling	9197	1:1000
P-CREB	Rabbit	Cell signaling	9198	1:1000
Tubulin	Rabbit	Cell signaling	2148	1:1000
MCAD	Mouse	Santa Cruz	sc-365030	1:1000
Total oxphos	Mouse	Cell signaling	110413	1:1000
Adora2a /Adenosine receptor A2a	Rabbit	Cell signaling	94871	1:1000

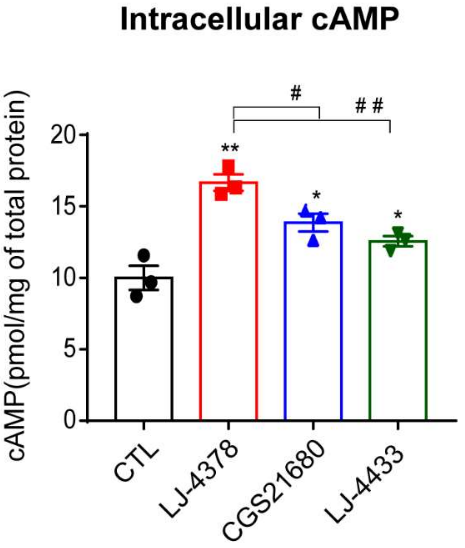

Supplementary Figure S1. Effects of LJ-4378 treatment on intracellular cAMP levels in brown adipocytes

Intracellular cAMP levels of brown adipocytes (BAs) treated with LJ-4378 (0.1 μM), $A_{2A}AR$ agonist (CGS21680, 0.1 μM), or A_3AR antagonist (LJ-4433, 0.1 μM) for 24 h (n = 3, means ± SEM, * $p < 0.05$, ** $p < 0.01$, # $p < 0.05$, ## $p < 0.01$). * : compared with CTL, # : compared with LJ-4378

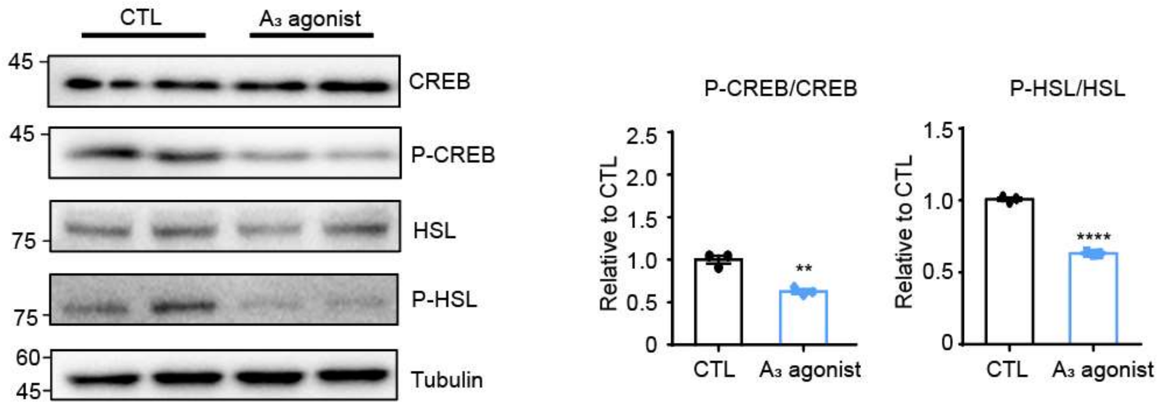

Supplementary Figure S2. Effects of A_3AR agonist (LJ-529) treatment on PKA signaling in brown adipocytes

Immunoblot analysis of brown adipocytes treated with A_3AR agonist (LJ-529, 0.1 μM) for 24h ($n = 3$, means ± SEM, ** $p < 0.01$, **** $p < 0.0001$).

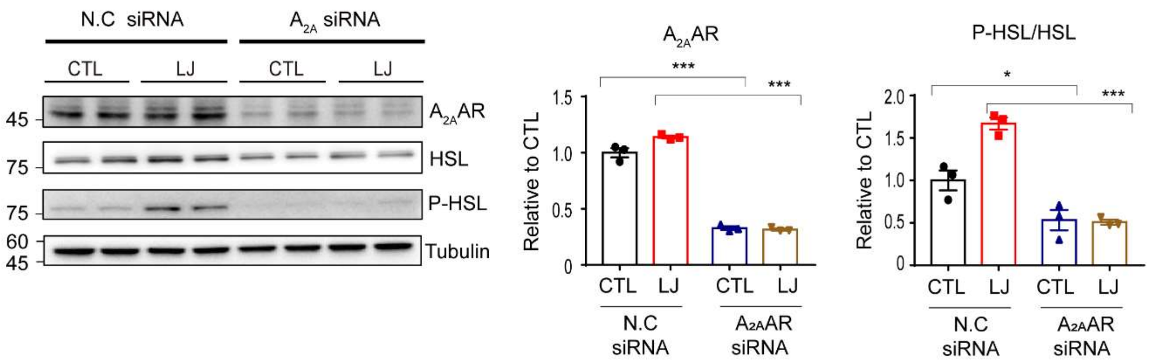

Supplementary Figure S3. Effects of $A_{2A}AR$ knockdown on LJ-4378-induced lipolysis of 3T3L1 adipocytes

Immunoblot analysis of effects of $A_{2A}AR$ knockdown on LJ-4378 (LJ)-induced phosphorylation of HSL (n = 3, means ± SEM, * $p < 0.05$, ** $p < 0.01$, *** $p < 0.001$).

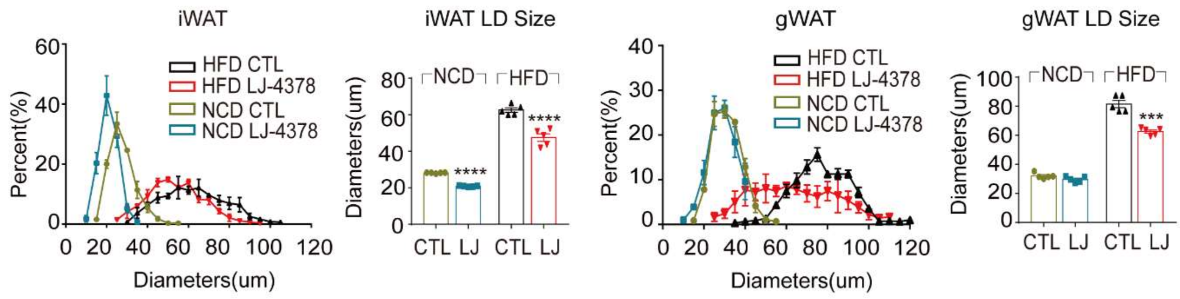

Supplementary Figure S4. Effects of LJ-4378 treatment on adipocyte size.

Quantification of adipocyte size in the images of H&E-stained paraffin sections of iWAT and gWAT of NCD- or HFD- fed mice treated with vehicle control (CTL) or LJ-4378 (LJ) ($n = 5$, means ± SEM, *** $p < 0.001$, **** $p < 0.0001$).

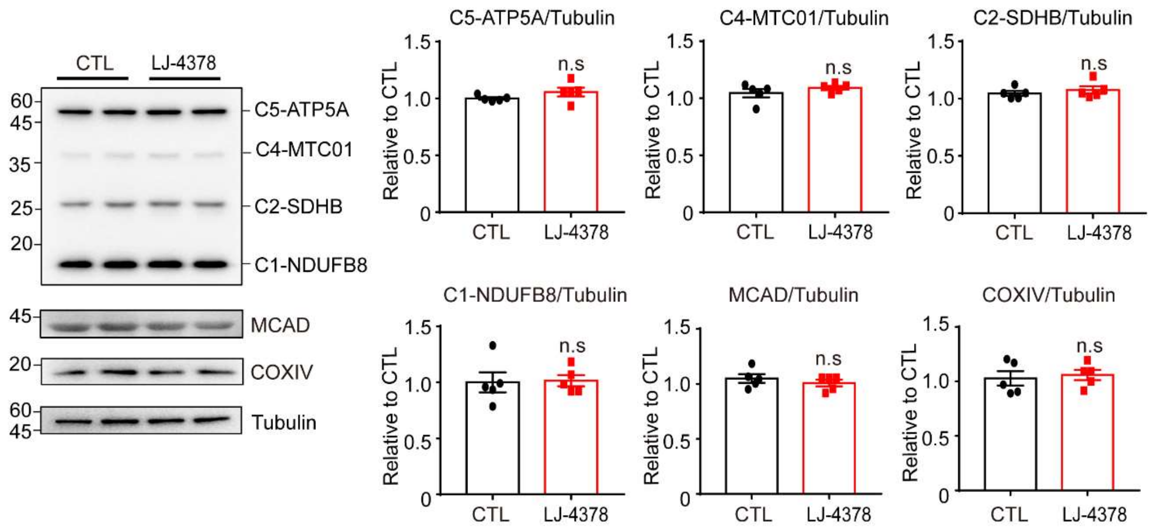

Supplementary Figure S5. Effects of LJ-4378 treatment on mitochondrial protein levels in skeletal muscle

Immunoblot analysis of mitochondrial proteins in gastrocnemius muscle of mice treated with LJ-4378 or vehicle controls (CTL) (i.p. 1 mg/kg for 10 days) n = 5.

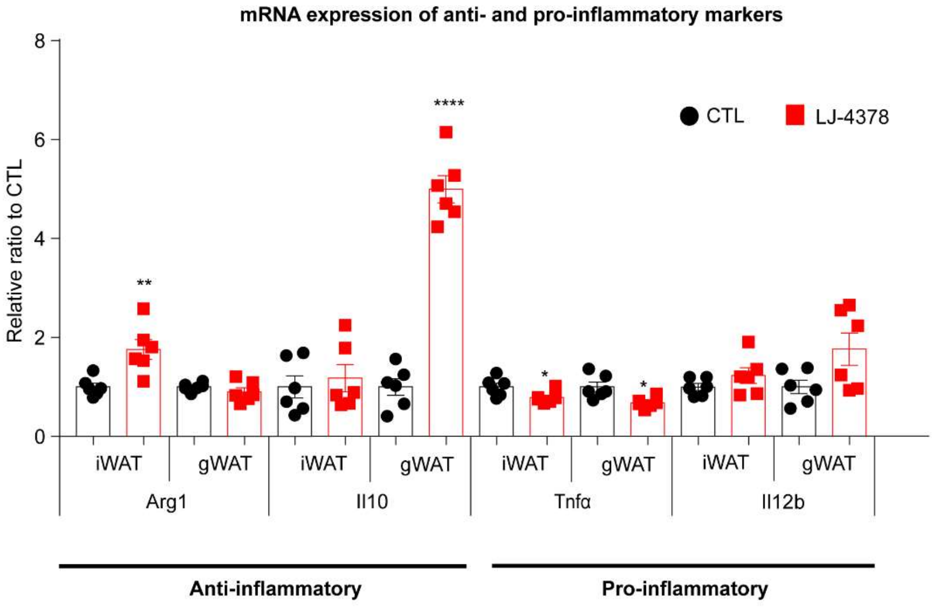

Supplementary Figure S6. Effects of LJ-4378 treatment on inflammatory marker expression levels in adipose tissue

High-fat diet (HFD)-fed mice were treated with LJ-4378 (LJ, 1 mg/kg) or vehicle controls (CTL) for 10 days. mRNA expression levels of pro-and anti-inflammatory markers were measured by qPCR analysis. (n = 6, means ± SEM, * $p < 0.05$, ** $p < 0.01$, **** $p < 0.0001$)
